# An Exploratory Investigation into the Effects of Methanol and Ethylene Glycol on the Growth and Development of Medaka (*Oryzias melastigma*) and Yellowstripe Goby (*Mugilogobius chulae*)

**DOI:** 10.3390/toxics14050380

**Published:** 2026-04-29

**Authors:** Zhenxiong Yang, Minxia Zhang, Tongfei Xu, Shasha Qi, Lu Tang, Juan Guo, Chuqian Lu, Shaobo Ma

**Affiliations:** 1 South China Sea Ecology Center, Ministry of Natural Resources, Guangzhou 510000, China; snrdfdcw@163.com (Z.Y.); 15817046041@163.com (T.X.); 13450491983@163.com (J.G.); luchuqan@163.com (C.L.); 2 Guangdong Provincial Field Observation and Research Station for Marine Ecosystem in Hanjiang River Estuary–Nanao Island Area, Shantou 515000, China; 3 China National Offshore Oil Corporation (China) Limited Beijing Research Center, Beijing 100016, China; zhangmx9@cnooc.com.cn (M.Z.); qishsh@conooc.com.cn (S.Q.); 4 College of Fisheries Sciences, Ludong University, Yantai 264025, China; tanglu17@mails.ucas.ac.cn

**Keywords:** marine fish, oxidative stress, acute toxicity, chronic toxicity, species specificity

## Abstract

This study aimed to investigate the acute and chronic toxic effects of two thermodynamic inhibitors (methanol and ethylene glycol) widely used in deep-sea oil and gas operations on two typical marine fish species, the medaka (*Oryzias melastigma*) and the yellowstripe goby (*Mugilogobius chulae*), to assess their potential ecological risks in marine environments. The 96-h median lethal concentration (LC_50_) was determined through acute toxicity tests. A 56-day chronic toxicity test was conducted to evaluate the effects on fish growth (body length) and the antioxidant defense system, specifically the activities of superoxide dismutase (SOD) and catalase (CAT). The results revealed marked species-specific differences. In terms of acute toxicity, medaka exhibited exceptionally high sensitivity to ethylene glycol (LC_50_ 15.77 g/L), while the yellowstripe goby showed greater tolerance (LC_50_ 22.17 g/L). Chronic exposure led to concentration-dependent growth inhibition in both species, and medaka showed significantly higher mortality than yellowstripe goby. Under methanol exposure, medaka exhibited significantly higher mortality (30–45%) than yellowstripe goby (5–20%). When exposed to ethylene glycol, medaka showed markedly high mortality (55–85%), while yellowstripe goby mortality remained below 15%. At the molecular level, both chemicals induced oxidative stress, but the response patterns of the antioxidant enzymes (SOD and CAT) were species-specific, indicating differences in toxic mechanisms and detoxification capacities. Methanol and ethylene glycol pose non-negligible ecotoxicological risks to marine fish, and the toxicity intensity is influenced by species specificity, exposure concentration, and the effectiveness of the antioxidant defense system. This study emphasizes that environmental risk assessments for such chemicals must fully account for species differences and sublethal effects, providing critical scientific evidence for formulating precise environmental safety standards for marine hydrocarbon exploitation.

## 1. Introduction

Methanol and ethylene glycol are widely used in deep-sea oil and gas operations to prevent the formation of methane clathrate hydrates and ice blockages within subsea pipelines [1,2,3]. Deep-sea environments are characterized by low temperatures and high pressures—conditions conducive to hydrate formation—during both drilling and production operations. The system pressure required to maintain flow from the seabed to the surface often exceeds the hydrate equilibrium pressure, thereby increasing the risk of hydrate nucleation and pipeline blockage [4]. In deepwater gas operations, thermodynamic inhibitors such as methanol or ethylene glycol are typically injected continuously during production to shift the hydrate phase equilibrium and mitigate formation risks [5]. This practice provides operational flexibility, including the ability to execute shut-in procedures while maintaining hydrate prevention. With the ongoing advancement of offshore oil and gas development, these two inhibitors are being released in increasing amounts in marine environments [2,3,6]. However, systematic field data on their environmental concentrations in globally distributed offshore production areas remain extremely scarce. To date, no comprehensive monitoring data have been reported to quantify the actual discharge levels or ambient concentrations of these two inhibitors in seawater or sediment in major oil-producing regions worldwide.

Research on the toxicity of methanol and ethylene glycol remains scarce and largely lacks data from marine species, as existing studies have primarily investigated freshwater fish [7,8]. Marine and freshwater environments differ significantly in key physicochemical factors such as salinity and hydrostatic pressure, which can alter the bioavailability and toxicokinetic processes of organic chemicals, leading to divergent toxic effects on aquatic organisms compared to freshwater systems [9]. Thus, employing marine fish models is essential for accurate risk assessment of two inhibitors in marine environments. The yellowstripe goby (*Mugilogobius chulae*) is a small benthic fish commonly found in brackish environments such as estuaries, coastal waters, and tidal river zones. Due to its small size, high fecundity, ease of laboratory maintenance, and sensitivity to pollutants, it is considered a suitable species for ecotoxicological studies [10]. Similarly, the marine medaka (*Oryzias melastigma*) is a pelagic fish also widely used in marine ecosystem toxicology [11,12]. Selecting these two species with distinct ecological niches (benthic vs. pelagic) enables the coverage of different marine micro-environments, making the toxicity assessment results more ecologically representative, and both are recognized as valuable indicator organisms for evaluating the two inhibitors’ multisystem toxicity under environmentally realistic salinity regimes.

In this study, we employed these two model marine fish species representing distinct ecological niches (water-column vs. benthic) to evaluate the acute and chronic toxicity effects of methanol and ethylene glycol. Notably, the test concentrations in this work correspond to localized oil-spill hotspot levels rather than routine environmental background concentrations, reflecting realistic high-pollution scenarios in offshore oil-producing areas. We determined the 96 h LC_50_ to characterize acute toxic effects, analyzed changes in body length and growth rate to reflect chronic growth inhibition, and detected the activities of hepatic SOD and CAT to explore oxidative stress responses, aiming to reveal the toxic mechanisms and species-specific differences in the two chemicals on the two marine fish, and further provide reliable experimental evidence for the ecological risk assessment and environmental safety management of methanol and ethylene glycol in marine environments.

## 2. Materials and Methods

### 2.1. Chemicals and Fish

Analytical grade methanol and ethylene glycol were obtained from the Solarbio Science & Technology Company (Beijing, China). The chemicals were administered in liquid form directly to the test medium, with their added volume converted to mass, and the treatment concentration expressed as mass per unit volume.

Yellowstripe goby (*Mugilogobius chulae*) and medaka (*Oryzias melastigma*) used in this study were obtained from the Guangdong Laboratory Animals Monitoring Institute, and maintained in a recirculating artificial seawater system (water exchange rate: 30% d^−1^) under controlled conditions: temperature 24 ± 1.0 °C, salinity 30 ± 1, and a 12 h: 12 h light-dark cycle. A total of 288 healthy adult individuals and 400 two-month-old juveniles of each fish were acclimated for 7 days prior to the formal experiment.

### 2.2. Experimental Design and Procedure

Five concentration groups for acute toxicity tests and four sublethal concentration groups for chronic toxicity tests were set for both methanol and ethylene glycol. In the acute exposure experiment, four parallel fresh seawater control groups were included, organisms were exposed to methanol or ethylene glycol across a range of five concentrations (2.5–25.0 g/L and 5.0–35.0 g/L, respectively). These concentration levels were chosen based on preliminary data from previous studies [7,8] and set relatively high to simulate locally elevated concentrations near leakage sources under offshore spill scenarios before full dilution in seawater. Accordingly, four sublethal concentrations (10%, 20%, 30%, and 40% of the LC_50_) were selected for the chronic exposure tests, with the methanol concentrations being 1.75 g/L (10%), 3.5 g/L (20%), 5.25 g/L (30%), and 7.0 g/L (40%), and the ethylene glycol concentrations being 1.6 g/L (10%), 3.15 g/L (20%), 4.7 g/L (30%), and 6.3 g/L (40%).

Throughout all experiments, water quality parameters (pH: 7.8 ± 0.2, temperature: 24 ± 1.0 °C, dissolved oxygen saturation: >90%) were maintained consistent with the fish acclimation conditions. For acute tests, six adult fish were randomly placed in each 2.5 L beaker, with four replicates per treatment. Mortality was recorded over 96 h without feeding to avoid interference from excretory products [13]. The median lethal concentration (LC_50_) for the acute tests was estimated using probit analysis based on 96 h cumulative mortality [14]. For the chronic tests, ten 2-month-old juvenile fish were randomly placed in each 2.5 L beaker, also with four replicates, and maintained for 56 days under a semi-static regime wherein half of the test water was replaced every two days to maintain stable chemical concentrations. Approximately 1 mL of hatched Artemia was supplied to each tank daily, and all feed was consumed within 10 min after administration. Fish were fed daily with commercially available hatched Artemia until 24 h before toxicity assessment. In both assays, food residues, feces, and dead fish were promptly removed to minimize interference.

### 2.3. Determination of Enzyme Activities and Body Length

The activities of superoxide dismutase (SOD) and catalase (CAT) in liver tissues were determined according to the methods described by previous studies [15,16]. For enzyme analysis, three fish were used from each concentration group, resulting in a total of 15 samples per fish species for both SOD and CAT assays. For morphometric measurements, three fish were randomly selected and measured from each treatment group. SOD activity was measured based on the inhibition of nitroblue tetrazolium (NBT) reduction by light at 450 nm. One unit of SOD activity (U) was defined as the amount of enzyme required to achieve 50% inhibition of NBT photochemical reduction. CAT activity was assessed by monitoring the decomposition of hydrogen peroxide (H_2_O_2_) at 240 nm for 1 min. One unit of CAT activity (U) corresponded to the amount of enzyme that catalyzed the decomposition of 50% of H_2_O_2_ in 60 s at 25 °C. The activities of both SOD and CAT are expressed per gram of wet tissue mass (U/g wet weight).

Body lengths of yellowstripe goby and medaka were measured initially on day 0 before exposure, and subsequently on days 14, 21, 28, 42, and 56. Individuals were photographed using a Canon EOS 5D Mark IV camera (Canon Inc., Tokyo, Japan). The images were subsequently analyzed using tpsUtil 1.7.6 and tpsDig 2.31 software [17] to obtain standardized morphometric data. The growth rate was calculated using the formula: Growth rate = (Final body length − Initial body length)/Initial body length × 100%.

### 2.4. Statistical Analysis

The 96-h median lethal concentration (LC_50_) for acute toxicity was determined using probit analysis implemented with the LD_50_ [14].

All data derived from chronic toxicity assays, including measurements of superoxide dismutase (SOD) activity, catalase (CAT) activity, and body length, were analyzed using GraphPad Prism (version 10.4.2 for Windows, GraphPad Software, Boston, MA, USA, www.graphpad.com). Prior to one-way ANOVA, the normality of distribution and homogeneity of variance of the data were verified. A one-way ANOVA was conducted on the data from each sampling interval (Days 14, 21, 28, 42, and 56) to assess statistically significant differences among the groups. Following a significant ANOVA result (*p* < 0.05), Dunnett’s multiple comparisons test was employed to specifically compare all treatment groups to the control.

## 3. Results

### 3.1. Acute Toxic Effects of Two Inhibitors on Yellowstripe Goby and Medaka

No mortality was observed in control groups, and cumulative mortality increased in a concentration-dependent manner for both inhibitors in acute toxicity tests. The 96-h LC_50_ values and 95% confidence intervals (CI) of methanol and ethylene glycol to yellowstripe goby and medaka were determined (Table 1). The 96-h LC_50_ value of methanol was similar between species, which was 17.42 g/L (95% CI: 14.85–20.44 g/L) for yellowstripe goby and 17.59 g/L (95% CI: 15.87–19.50 g/L) for medaka. In contrast, ethylene glycol exhibited markedly different toxicity between the two species, with the 96-h LC_50_ being 22.17 g/L (95% CI: 18.91–25.99 g/L) for yellowstripe goby and 15.77 g/L (95% CI: 13.55–18.36 g/L) for medaka.

### 3.2. Chronic Toxicity Effects of Two Inhibitors on the Mortality and Growth of Medaka and Yellowstripe Goby

The mortality rates of yellowstripe goby and medaka following methanol exposure are summarized in Table 2. No mortality was observed in any control group during the 56-day chronic exposure.

**Figure 1:**
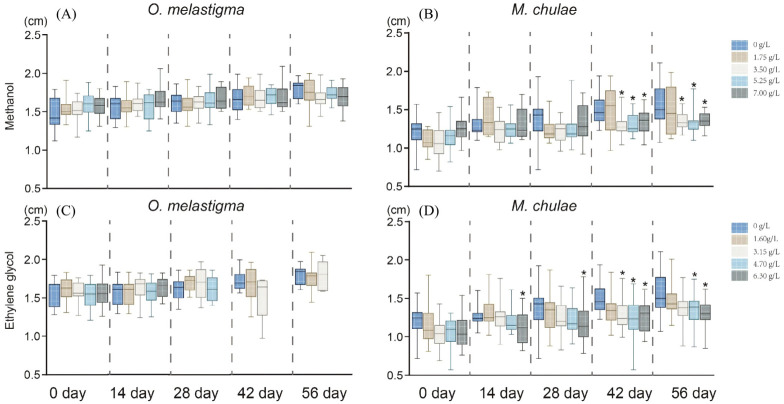
Concentration-dependent changes in body length and growth rate of *O. melastigma* (**A**,**C**) and *M. chulae* (**B**,**D**) after 56-day chronic exposure to methanol (**A**,**B**) and ethylene glycol (**C**,**D**). Significant differences compared with the control group are indicated by * *p* < 0.05.

Under methanol exposure, medaka exhibited considerable sensitivity, showing mortality rates ranging from 30% to 45% across all exposure concentrations. In contrast, the highest mortality observed in yellowstripe goby did not exceed 20%. Both species also showed noticeable changes in body length and growth rate under higher methanol concentrations (Figure 1A). Medaka body length showed minor reductions relative to the control after 56 days of exposure, though these differences were not statistically significant (*p* > 0.05). Specifically, the growth rate of medaka decreased in a concentration-dependent manner: the control group exhibited a growth rate of 21%, while rates in the 1.75, 3.5, 5.25, and 7.0 g/L treatment groups declined to 14%, 10%, 9%, and 8%, respectively.

In yellowstripe goby, a significant reduction in body length was observed from day 42 onward in the high-concentration group (3.5, 5.25 and 7.0 g/L, *p* < 0.05) (Figure 1B). Notably, the growth rates of goby across treatment groups remained relatively stable, with values of 29%, 30%, 31%, 28%, and 23% for the control, 1.75, 3.5, 5.25, and 7.0 g/L groups, respectively.

Similar trends were observed in ethylene glycol exposure (Table 2). Medaka demonstrated strikingly high mortality, ranging from 55% to 85% across concentrations. In yellowstripe goby, however, mortality remained comparatively low, not exceeding 15%. Body length was also affected under ethylene glycol treatment (Figure 1C). By day 42, medaka in high-concentration groups (3.15–6.3 g/L) exhibited mortality between 75% and 85%, indicating severe toxicity. The low-concentration group (1.60 g/L) showed no significant difference in body length compared to the control (*p* > 0.05). Growth rate analysis further supported these observations: medaka exhibited a pronounced reduction in growth rate across all ethylene glycol concentrations, with values of 21% (control), 11% (1.6 g/L), 13% (3.15 g/L), 14% (4.7 g/L), and 16% (6.3 g/L), indicating disrupted growth despite the non-significant change in absolute length (Table 3). Although mortality in yellowstripe goby remained below 15% throughout the experiment, a statistically significant reduction in body length was detected from day 42 onward in groups exposed to 3.5–7.0 g/L ethylene glycol (*p* < 0.05) (Figure 1D). Notably, the growth rate of yellowstripe goby displayed a clear concentration-dependent decline, with values of 29% (control), 36% (1.6 g/L), 28% (3.15 g/L), 20% (4.7 g/L), and 13% (6.3 g/L).

### 3.3. Effects on Hepatic Antioxidant Defense System

In our assessment of key enzyme activities within the antioxidant defense system, we observed significant effects (*p* < 0.01) of methanol and ethylene glycol exposure concentration on the activities of SOD and CAT in the liver tissues of the two fish species.

**Figure 2:**
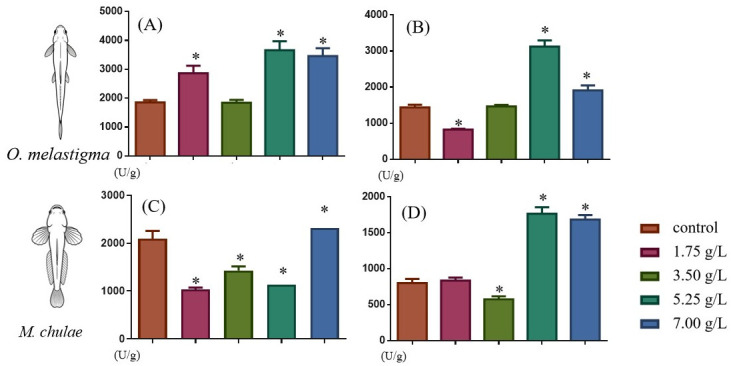
Concentration-dependent changes in hepatic superoxide dismutase (SOD, (**A**,**C**)) and catalase (CAT, (**B**,**D**)) activities of *O. melastigma* (**A**,**B**) and *M. chulae* (**C**,**D**) after 56-day chronic methanol exposure. Data are presented as mean ± SD (*n* = 3), with significant differences compared to the control group indicated by * *p* < 0.05.

Under methanol exposure, the activities of SOD and CAT exhibited concentration-dependent and species-specific responses in both medaka and yellowstripe goby (Figure 2), with SOD activity at 7 g/L, which was significantly higher than the control in both species (*p* < 0.05). However, a markedly divergent response occurred at lower concentrations: in yellowstripe goby, SOD activity was significantly suppressed relative to the control across all lower concentrations (1.75, 3.5, and 5.25 g/L, *p* < 0.05); in contrast, medaka showed a significant increase in SOD at 1.75 and 5.25 g/L (*p* < 0.05), while activity remained comparable to the control at 3.5 g/L (*p* > 0.05). CAT activity in both species was significantly elevated at higher methanol concentrations (5.25 and 7 g/L) compared to the control (*p* < 0.05). Species-specific differences were observed at lower concentrations: in medaka, CAT activity was significantly reduced at 1.75 g/L (*p* < 0.05) and slightly increased at 3.5 g/L (*p* > 0.05), whereas in yellowstripe goby, activity was slightly elevated at 1.75 g/L (*p* > 0.05) and significantly reduced at 3.5 g/L (*p* < 0.05).

**Figure 3:**
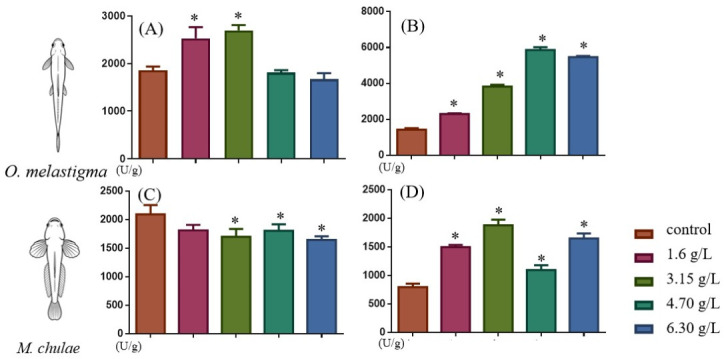
Concentration-dependent changes in hepatic superoxide dismutase (SOD, (**A**,**C**)) and catalase (CAT, (**B**,**D**)) activities of *O. melastigma* (**A**,**B**) and *M. chulae* (**C**,**D**) after 56-day chronic ethylene glycol exposure. Data are presented as mean ± SD (*n* = 3), with significant differences compared to the control group indicated by * *p* < 0.05.

Under ethylene glycol exposure, the responses of SOD and CAT activities exhibited distinct patterns between medaka and yellowstripe goby (Figure 3). For SOD activity, species-specific responses were observed: in medaka, SOD activity was significantly increased at 1.6 and 3.15 g/L (*p* < 0.05), while no significant differences were detected at higher concentrations (4.7 and 6.3 g/L, *p* > 0.05); in contrast, yellowstripe goby showed a consistent reduction in SOD activity across all concentration groups, and this decrease was statistically significant at 3.15, 4.7, and 6.3 g/L compared to the control group (*p* < 0.05). CAT activity was significantly elevated across all tested concentrations (1.6, 3.15, 4.7, and 6.3 g/L) in both species compared to the control group (*p* < 0.05).

## 4. Discussion

### 4.1. Interspecific Differences in Acute Toxicity

The 96 h LC_50_ values of methanol for the two marine fish species in this study (approximately 17.5 g/L) are similar to bluegill sunfish (*Lepomis macrochirus*; 15.4 g/L), but notably lower than those of rainbow trout (*Oncorhynchus mykiss*; 20.1 g/L) and fathead minnow (*Pimephales promelas*; 28.1–294 g/L) [18,19]. Notably, the test concentrations selected in this study simulate pollutant levels at localized oil-spill hotspots instead of routine environmental background levels, reflecting realistic high-pollution exposure scenarios in contaminated marine areas. This difference may be attributed to the distinct physiological characteristics between marine and freshwater fish, as the high salinity in marine environments could increase the bioavailability of methanol and thus enhance its toxic effects [20]. Ethylene glycol has generally been regarded as a substance of low toxicity to aquatic organisms in previous ecotoxicological studies [3]. However, the results from the present investigation reveal significant interspecific differences in sensitivity: the medaka demonstrated notably high susceptibility to ethylene glycol, with a 96-h LC_50_ of 15.77 g/L, which was approximately 2.6 times lower than that of rainbow trout (*O. mykiss*; 41 g/L) and substantially lower than those of juvenile fathead minnow (*P. promelas*; 53 g/L) and bluegill sunfish (*L. macrochirus*; >111.3 g/L) [1,21,22]. The high sensitivity of medaka to ethylene glycol may be related to its relatively weak metabolic capacity for ethylene glycol metabolites (glycolic acid and oxalic acid) compared to other fish species [8,23,24].

### 4.2. Chronic Growth Inhibition Induced by Methanol and Ethylene Glycol

Our findings indicated that chronic exposure to methanol resulted in concentration-dependent growth reduction in both medaka and yellowstripe goby, with the latter exhibiting significant growth suppression at high concentrations. Moreover, a marked interspecific difference in chronic mortality was observed: medaka showed considerable sensitivity, with mortality rates ranging from 30% to 45%, whereas in yellowstripe goby, mortality did not exceed 20%, indicating a higher tolerance to methanol toxicity. Thos aligns with previous research reporting that even low concentrations of methanol can induce growth inhibition and reproductive impairment in tilapia [7]. Although studies on metabolic disturbances induced by methanol in fish remain limited, it is well-established that chronic methanol exposure can lead to metabolic disorders, reduced feeding efficiency, and consequent impairments in fish growth [25,26].

Similarly, the present study also indicated that ethylene glycol exposure significantly impacts body length and growth rates in both medaka and the yellowstripe goby, though with notable interspecific differences in susceptibility and physiological response. In medaka, ethylene glycol induced severe toxicity at higher concentrations (3.5–7.0 g/L), manifesting as significantly elevated mortality (75–85% by day 42) and a pronounced reduction in growth rate across all treatment groups. Notably, even the lowest concentration (1.6 g/L) resulted in reduced growth rates compared to the control, suggesting that sublethal metabolic inhibition may occur prior to morphological manifestations. This growth suppression likely reflects underlying disruptions in energy metabolism and protein synthesis, consistent with known toxic effects of ethylene glycol metabolites such as glycolic and oxalic acid, which can impair cellular respiration and nutrient assimilation [8,23,24]. In contrast, the yellowstripe goby exhibited markedly lower mortality (<15%), supporting its relatively higher tolerance to ethylene glycol. However, a significant reduction in body length was observed from day 42 onward under higher concentrations (3.5–7.0 g/L), accompanied by a clear concentration-dependent decline in growth rate. This pattern implies that while the yellowstripe goby possesses physiological or behavioral adaptations that enhance survival, which possibly occur through more efficient detoxification pathways or improved oxidative stress management, it remains vulnerable to chronic growth impairment under sustained chemical stress.

### 4.3. Species-Specific Antioxidant Responses and Toxic Mechanisms

The divergence in responses between these two species may be attributed to differences in ethylene glycol and methanol metabolism and elimination capacity. The yellowstripe goby’s benthic ecology might have driven the evolution of enhanced enzymatic activity or renal clearance mechanisms that mitigate acute toxicity yet do not fully prevent chronic growth effects [10]. By comparison, medaka appear less capable of detoxifying ethylene glycol or its metabolites, resulting in both higher mortality and more severe growth retardation.

Hepatic metabolism of foreign compounds often generates reactive oxygen species (ROS), and SOD and CAT belong to the first line of defense for eliminating ROS and can be inhibited by excess ROS in turn [27,28,29]. Similar nonlinear, biphasic responses of SOD and CAT have been widely reported in fish exposed to heavy metals, organic pollutants, and triclosan (TCS) [30,31]: in zebrafish (*Danio rerio*), these enzymes exhibited concentration-dependent non-monotonic changes, including initial induction at low concentrations, weakened induction or inhibition at intermediate concentrations, and decline or compensatory upregulation when exceeding a toxic threshold. Specifically, low concentrations of TCS may more effectively activate the antioxidant enzyme system, while high concentrations may affect oxidative stress responses through other pathways, with CAT activity showing an “inverted U-shaped” nonlinear dose–response in chronic exposure [30,31]. Under methanol exposure, the significant induction of CAT and SOD activities at higher concentrations in both species strongly suggests a compensatory upregulation of the antioxidant defense system in response to elevated reactive oxygen species (ROS) generation. However, the suppression of SOD activity across intermediate concentrations in yellowstripe goby, contrasted with the general induction or maintenance of activity in medaka, may indicate a lower threshold for oxidative insult or a less resilient antioxidant capacity in the yellowstripe goby. This finding aligns with the higher mortality observed in medaka compared to yellowstripe goby, suggesting that while the medaka suffers greater lethal toxicity, its antioxidant enzymatic response remains more robust at lower challenge levels.

The response to ethylene glycol presented a notably different pattern. The consistent and significant induction of CAT activity across all concentrations in both species points to a potent and pervasive generation of hydrogen peroxide, a key substrate for CAT. This uniform CAT response suggests that the pathway of ethylene glycol toxicity, potentially via its metabolite glycolate [8,23,24], reliably induces this particular oxidative challenge. The stark contrast in SOD activity between the species—induced in medaka at lower concentrations versus significant suppression in yellowstripe goby—further underscores fundamental differences in their handling of superoxide anion radicals. Such interspecific differences in antioxidant sensitivity and biphasic enzyme regulation are consistent with observations in other fish species [30,31], where SOD and CAT exhibit typical nonlinear patterns such as inhibition followed by induction or attenuated induction with increasing contaminant levels. The suppression of SOD in yellowstripe goby could reflect enzyme inhibition, a failure to upregulate enzyme synthesis under sustained stress, or even more severe oxidative damage to the enzymatic machinery itself [32,33]. This pronounced inhibition is also evidenced by the significant growth reduction observed in yellowstripe goby under ethylene glycol exposure, as accumulated superoxide radicals can cause substantial cellular damage to proteins, given that the accumulation of superoxide radicals can inflict severe cellular damage on proteins, lipids and nucleic acids, which in turn impairs the normal growth and development of the fish.

## 5. Conclusions

The distinct antioxidant response profiles between medaka and yellowstripe goby exposed to methanol and ethylene glycol provide compelling evidence for species-specific toxicological mechanisms, which are likely rooted in variations in metabolic pathways, antioxidant system efficiency, and inherent tolerance to oxidative stress of the two fish species. Both methanol and ethylene glycol can cause concentration-dependent growth inhibition and oxidative stress in medaka and yellowstripe goby, and medaka has a higher sensitivity to the two chemicals than yellowstripe goby in terms of acute and chronic mortality. Our findings underscore the importance of considering species-specific responses and sublethal effects in ecological risk assessments for these common organic pollutants in deep-sea oil and gas operations, and provide critical scientific evidence for formulating precise environmental safety standards for marine hydrocarbon exploitation.

## Figures and Tables

**Table 1:** The 96 h LC_50_ of methanol and ethylene glycol to *M. chulae* and *O. melastigma*.

Chemicals	Species	96 h LC50 (g/L)	95% CI (g/L)
Methanol	*O. melastigma*	17.59	15.87–19.50
	*M. chulae*	17.42	14.85–20.44
Ethylene glycol	*O. melastigma*	15.77	13.55–18.36
	*M. chulae*	22.17	18.91–25.99

**Table 2:** Mortality rates of *M. chulae* and *O. melastigma* under 56-day chronic exposure to methanol and ethylene glycol.

Methanol			Control	1.75 g/L	3.5 g/L	5.25 g/L	7 g/L
	*O. melastigma*	Initial Number	20	20	20	20	20
		Final Number	20	14	11	11	12
		DR (Death rate)	0%	30%	45%	45%	40%
	*M. chulae*	Initial Number	20	20	20	20	20
		Final Number	20	17	18	16	19
		DR (Death rate)	0%	15%	10%	20%	5%
Ethylene glycol			Control	1.6 g/L	3.15 g/L	4.7 g/L	6.3 g/L
	*O. melastigma*	Initial Number	20	20	20	20	20
		Final Number	20	9	5	3	3
		DR (Death rate)	0%	55%	75%	85%	85%
	*M. chulae*	Initial Number	20	20	20	20	20
		Final Number	20	18	17	19	19
		DR (Death rate)	0%	10%	15%	5%	5%

**Table 3:** Growth rate of *M. chulae* and *O. melastigma* under 56-day chronic exposure to methanol and ethylene glycol.

Methanol			Control	1.75 g/L	3.5 g/L	5.25 g/L	7 g/L
	*O. melastigma*	Initial Length	1.48	1.53	1.55	1.59	1.58
		Final Length	1.79	1.74	1.70	1.73	1.70
		LGR (Length gain rate)	21%	14%	10%	9%	8%
	*M. chulae*	Initial Length	1.20	1.14	1.05	1.06	1.05
		Final Length	1.55	1.48	1.38	1.36	1.29
		LGR (Length gain rate)	29%	30%	31%	28%	23%
Ethylene glycol			Control	1.6 g/L	3.15 g/L	4.7 g/L	6.3 g/L
	*O. melastigma*	Initial Length	1.48	1.61	1.58	1.52	1.55
		Final Length	1.79	1.79	1.78	1.73	1.79
		LGR (Length gain rate)	21%	11%	13%	14%	16%
	*M. chulae*	Initial Length	1.20	1.10	1.06	1.15	1.27
		Final Length	1.55	1.50	1.36	1.38	1.43
		LGR (Length gain rate)	29%	36%	28%	20%	13%

## Data Availability

The original contributions presented in this study are included in the article. Further inquiries can be directed to the corresponding author.
